# A Sensitive Fibre Optic Probe for Autofluorescence Spectroscopy of Oral Tongue Cancer: Monte Carlo Simulation Study

**DOI:** 10.1155/2020/1936570

**Published:** 2020-04-08

**Authors:** Haneen Shhadeh, Wesam Bachir, George Karraz

**Affiliations:** ^1^Biomedical Photonics Laboratory, Department of Laser Physics and Technology, Higher Institute for Laser Research and Applications, Damascus University, Damascus, Syria; ^2^Faculty of Informatics Engineering, Al-Sham Private University, Damascus, Syria

## Abstract

The objective of this paper is to determine the best optical probe configuration that would help to detect neoplastic lesions in oral tongue epithelial tissue. Three geometrical configurations are investigated. The first one is a single-fibre probe with different fibre diameters. The second one is a multitilted fibre probe that employs different tilting angles for emission and collection fibres. While the third one is a multidiameter probe that employs different fibre diameters and distances between the emission and the collection fibres. All probes were evaluated for their depth-limited sensitivity in the epithelium layer of the tongue. Probes that showed efficient sensitivities were then compared for their fluorescence intensities acquired from both tissue types. The sensitivity for the first two types of probes was found to be roughly comparable. However, the differentiation capability of the multitilted fibre probe between dysplastic and healthy tissue was found to be noticeably larger by 30% of that of the single-fibre probe. The third type showed more sensitivity to fluorescence emerging from deeper layers. Finally, the proposed configuration is presented and proved to achieve higher sensitivity for both superficial and deep layers.

## 1. Introduction

Oral cancer is a serious health problem worldwide [1, 2]; early detection of these cancers can greatly reduce morbidity rates [3]. Neoplasia can occur in any location in the oral cavity; however, oral cancer is more likely to develop on the tongue surface [4]. Recently, optical spectroscopy has been a promising modality for rapid and noninvasive diagnosis of oral cancer [5]. Nonetheless, detecting precancerous lesions in the oral cavity can be a challenging task, since it is hard to clinically differentiate between normal lesions and malignant ones [6], the determination process could involve several biopsies as well and it depends on the expertise of the clinician who is in charge. Recently, autofluorescence spectroscopy has emerged as an effective and noninvasive technique to discriminate precancer in human oral tissue [7]. It has a capability to detect alternations in tissue optical properties during malignant transformation [8]. It has been proved that detecting neoplastic transformation in oral epithelial tissue is associated with morphological and structural changes that alter the optical properties of the tissue [9–11]. Furthermore, these alterations include increasing in superficial layer fluorescence intensity and decreasing in deeper layer fluorescence intensity [12].

However, one of the main drawbacks of the fluorescence spectroscopy is that the fluorescent signal is highly dependent on fluorophore distribution within the tissue, optical properties, and on the probe geometry [13]. Different groups have investigated different geometrical designs with different tissues [14, 15]. Multitilted fibre (MTF) probes were extensively studied for biomedical optical spectroscopy [8]. A single-fibre (SF) probe geometry has gained much interest for fluorescence measurements as well [16].

In this paper, we compare and contrast the two probe geometries that are commonly used for autofluorescence spectroscopy; in addition, we proposed a third geometry that implements Multidiameter of the emission and collection fibres (MDF) with the aim to detect dysplastic progression in the tongue epithelial tissue at an early stage. Finally, the sensitivity of the proposed probe is analysed and discussed.

## 2. Materials and Methods

For this purpose, five-layer epithelial tongue tissue models were used. The first model represents the normal tissue and the second one represents the mild dysplastic transformation in malignant epithelial tissue of the tongue. As found previously, the fluorophore distribution in the layers is depth dependent [17, 18]. Here, we describe it as follows: A superficial layer dominated by keratin fluorophore, an intermediate layer by FAD fluorophore, a basal layer dominated by NADH fluorophore, a superficial stroma dominated by collagen, and a deep stroma that is considered too deep to model a semi-infinite medium is also dominated by collagen [19].

The optical properties for each layer including absorption coefficient (*μ*_*a*_) and scattering coefficient (*μ*_*s*_) for excitation and emission wavelengths (350 and 420 nm) were taken from the literature [19].  Table 1 lists the values for both tissue models.

**Figure 1:**
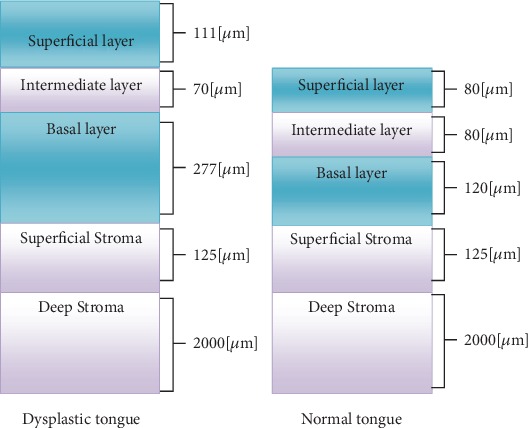
Dysplastic and normal tongue layers.

Figure 1 shows a comparison between the normal and dysplastic layers.

Previous works on the origin of fluorescence in the models given above reported that dysplastic tissue showed increased fluorescence intensity in superficial layers and decreased fluorescence intensity in underlying layers. Hence, a fibre optic probe that can separately interrogate the superficial layer would significantly enhance the efficiency of the fibre probe in identifying carcinoma development in biological tissue [6, 13].

As shown in Table 1 and Figure 1, all layers show fluorescence intensity differences when cancerous alternation occurs except for the basal layers that show the same values for both tissue types. Accordingly, its participation in discriminating normal and dysplastic tissues could be neglected.

The targeted probe in this study was aimed at achieving high sensitivity for both superficial and stroma layers of normal and dysplastic tongue sites by using the same configuration for both of them.

To do this, one boundary between the superficial and stroma layers had to have the same value for normal and dysplastic tissues.

The depth 280 *μ*m, which is the sum of depths of the three superficial layers in the normal tongue model, was chosen because it works well for normal tissue and includes only some of the basal fluorescence from dysplastic tissue that was said to be neglected as it does not change with cancerous alternations.

All geometries tested below were evaluated according to their sensitivity to fluorescence above and beneath this boundary.

**Figure 2:**
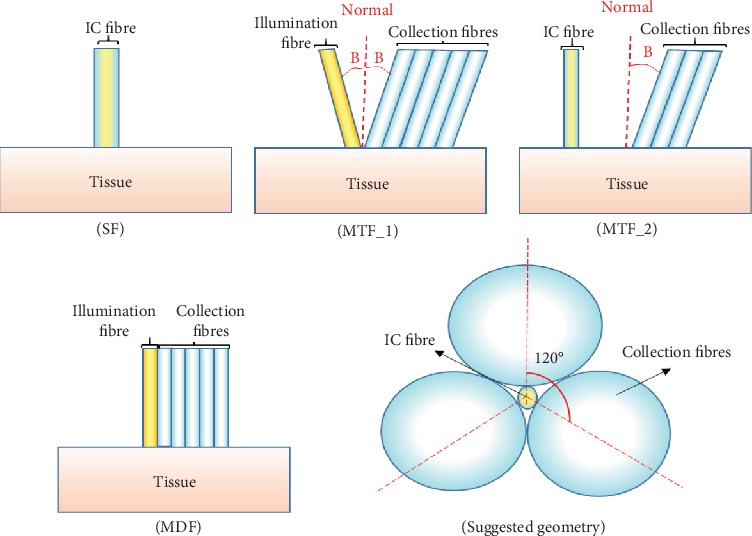
All probe geometries.

The studied geometries are displayed in Figure 2.

- Single-fibre probe (SF): where there is only one fibre positioned vertically on the tissue and performs both the illumination and collection processes (IC fibre). The fibre diameter was varied in the range of 100-900 *μ*m in increments of 100 *μ*m. With this configuration, it can be noticed that both the illumination and collection cones are totally overlapped.
- Multitilted fibre probe (MTF_1): one illumination fibre and five collection fibres that are all set at 100 *μ*m and tilted form the normal of the surface. The tilting angle (*B*) was varied in the range of (25°-50°) with a step of (5°).
- Multitilted fibre probe (MTF_2): similar to the previous one, but here the illumination fibre was fixed vertically on the tissue and was used as an illumination and collection fibre (IC fibre). In addition, the first two collection fibres were removed and the rest three ones were kept at their original positions and fixed at a tilting angle of 25° only.
- Multidiameter fibre probe (MDF): one illumination fibre set at 200 *μ*m and five collection fibres that are varied in diameter in the range of 200-800 *μ*m and in increments of 200 *μ*m.
- Suggested fibre: one illumination and collection fibre (IC fibre) that is set at 100 *μ*m, and three other collection fibres that surround the first one and are set at 600 *μ*m.

In all configurations, fibres were considered adjacent to the tissue as there was no air gap in between and the fibre used for illumination and collection at the same time was referred to as (IC).

In addition, the numerical aperture (NA) and the core refractive index for all geometries were 0.5 and 1.466, respectively.

The refractive index for all layers was considered constant and equal to 1.4. As for the anisotropy factor, it was set at 0.94 for the superficial, intermediate, and basal layers and 0.84 for the two stromal layers [8].

A Monte Carlo- (MC-) based software program, written in MATLAB (MATLAB 2015, MathWorks Inc., USA), was used to simulate photon propagation in the selected tissue models through a given fibre probe geometry; 10^6^ photons were used in each simulation [20]; the error in results was <<0.01, thus the number of photon used for simulation is justified. Photons were launched in a random uniform distribution within fibre diameter and numerical aperture [21].

Sensitivity of the fibre probe can be defined as the ratio of fluorescence photons detected from a certain layer to the total number of fluorescence photons detected. It can be expressed as follows:

(1) $$\begin{array}{c} S_{s}=\frac{\text{Number of fluorescence photons collected from suprficial layers}}{\text{The total number of fluorescencce photon collected}}*100\%, \end{array}$$

(2) $$\begin{array}{c} S_{d}=\frac{\text{Number of fluorescence photons collected from stromal layers}}{\text{The total number of fluorescence photon collected}}*100\%, \end{array}$$

where *S*_*s*_ is the sensitivity to the superficial layers and *S*_*d*_is the sensitivity of the stromal layers.

As shown in Table 1, the fluorescence in the first two superficial layers and the first stromal layers varies between the normal and dysplastic tissues. Thus, a configuration that can differentiate between fluorescence photons originating from these layers in both tissue cases could help in detecting precancerous lesions.

**Figure 3:**
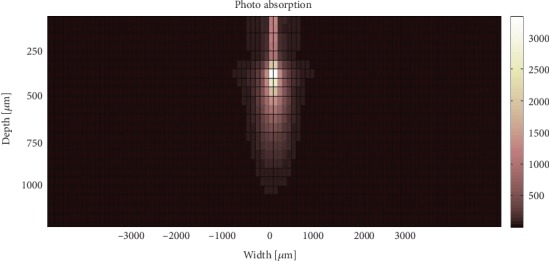
Absorption positions within normal tissue model when using SF geometry with 100 *μ*m fibre diameter.

**Figure 4:**
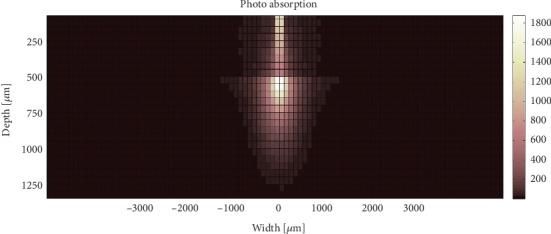
Absorption positions within dysplastic tissue model when using SF geometry with 100 *μ*m fibre diameter.

As an illustrative example, Figures 3 and 4 show the light penetration in dysplastic and normal tissue models using SF geometry fibre with a diameter of 50 *μ*m.

Monte Carlo simulation is described in detail in previous works, particularly [23]. However, a brief description is given here. The simulation depends on launching many photons simultaneously as packets, to minimize the number of photons required to achieve sufficient accuracy for its calculation.

1. Packet of photons is given an initial weight of one, initial direction, position within the fibre diameter and step size
2. The packet moves by the predetermined steps size. Then, part of its weight is absorbed in the position it reached (absorption event)
3. The absorbed weight value is calculated depending on the optical properties of the layer the packet is in. Then, the weight of the packet is updated by subtracting the absorbed weight
4. New directions and step size are then calculated and assigned to the packet that will move accordingly to its new location
5. Steps 2, 3, and 4 are repeated until the packet exits the tissue or is being collected by one of the collection fibres or its weight drops under a certain limit
6. Another packet is then launched and traced

To simulate fluorescence, a calculation is made after each absorption event to determine whether this packet will produce a fluorescence packet or not. If so, simulation of the original packet is ceased, the absorbed weight is assigned to the fluorescence packet as an initial weight, the step size and directions are calculated according to the layer optical properties associated with the emission wavelength, and the simulation continues the same way as for the original packet (steps 2, 3, 4, and 5).

After completing the simulation of the fluorescence packet, the original packet continues forming the position it was in. All optical phenomena such as reflection and transmission are taking into consideration this simulation.

## 3. Results and Discussion

All the geometries were tested on the dysplastic tissue model and evaluated for their sensitivity to the superficial and stroma layers. The geometries that showed efficient sensitivity were then tested on normal tongue model and the results were then compared.

### 3.1. SF Geometry

**Figure 5:**
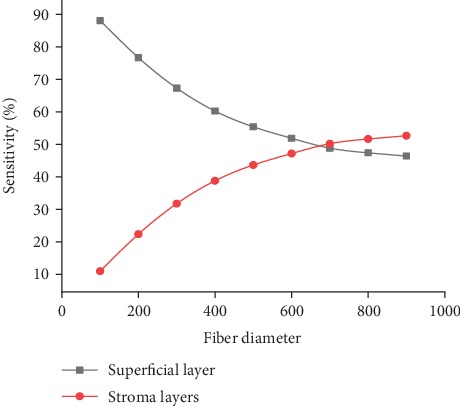
Sensitivity to superficial and deep layers using the SF probe.

Results shown in Figure 5 demonstrate that increasing the fibre diameter results in increasing the number of the detected fluorescence photons. However, the sensitivity to superficial layers is inversely proportional to the fibre diameter. The sensitivity was found to be highest (88%) when using a 100 *μ*m diameter fibre, while the stromal sensitivity peeked at 55% when using a 1000 *μ*m diameter fibre. This is due to the fact that the larger the diameter, the larger the surface of contact with the tissue is. Accordingly, more photons can be collected especially the ones that travel away from the illumination source and propagate to deeper depths. On the contrary, the small diameter of illumination means that photons will propagate in depths closer to the source. Thus, the fibre will collect more of them than other photons.

These results are in agreement with a previous study [9] that also suggests that the larger the diameter, the larger the probing depth is (the depth from which 80% of fluorescence occurs), so for small diameters the photons will propagate in the shallow depths and accordingly will be more likely to be collected by the fibre, whereas for the larger diameters, the penetration depth increases which means that the fluorescence will occur in deeper depths and the photons will be more likely to separate away from the source and to be collected at larger distances from the place they were originally launched from.

Therefore, this geometry works best for detecting fluorescence originating from superficial layers especially when using the 100 *μ*m diameter fibre, but it does not show such capability regarding the stromal layers.

### 3.2. MTF_1 Probe

**Figure 6:**
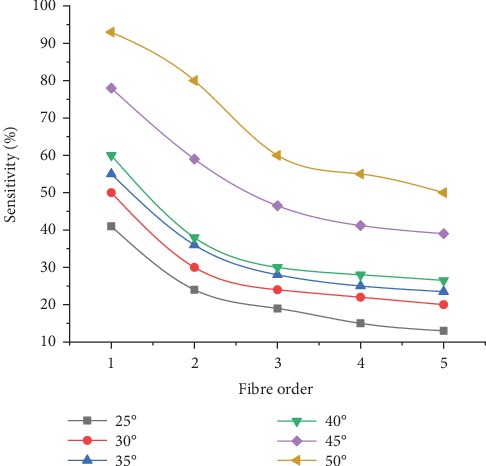
Sensitivity to superficial layers using MTF_1.

MC simulation results showed that increasing the tilting angle increased the superficial sensitivity. Also, increasing the distance between the illumination and collection fibres decreased the number of collected fluorescence photons and decreased the superficial sensitivity. As can be seen in Figure 6, the best superficial sensitivity value in this configuration was found to be approximately 93%. This value corresponded to the first collection fibre tilted at 50°. The reason for that could be due to the overlapping of the illumination and collection cones in the superficial layers and the increased contact surface of the illumination fibre with the tissue surface.

**Figure 7:**
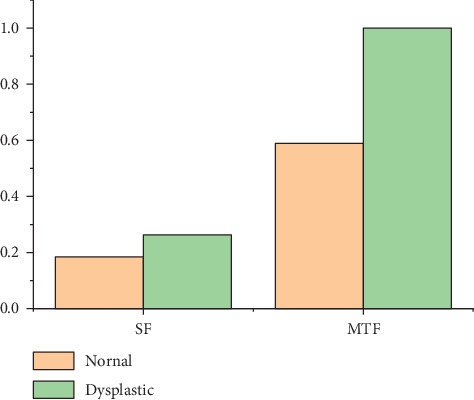
Normalized total number of fluorescent photons detected from normal and dysplastic tongues using SF and MTF_1 probes at 50°.

The sensitivity to stromal layer fluctuated in the range 80–86% in the last three fibres at *B* = 25°, but the number of photons detected was too small. With respect to the discriminating capability of the fibre probes mentioned above at *B* = 50°, the number of fluorescence photons detected from healthy and dysplastic oral tongue tissues using the first two fibre probes were assessed as shown in Figure 7. The results show that the number of fluorescence photons detected from the dysplastic tongue using the MTF probe was found to be 1.7 times higher than that emerged from the normal tongue. Also, the number of fluorescence photons detected from the dysplastic tongue using SF was found to be 1.4 times higher than that collected from the normal tongue. Consequently, it can be concluded that the differentiating capability of the MTF probe is higher than that of the SF probe by 30%. This distinct difference in performance between the probes may be attributed to the increased contact surface between the fibre and the tissue and numerical aperture of the MTF probe geometry (due to its bevel ends) compared to those in the SF probe.

### 3.3. MTF_2 probe

**Figure 8:**
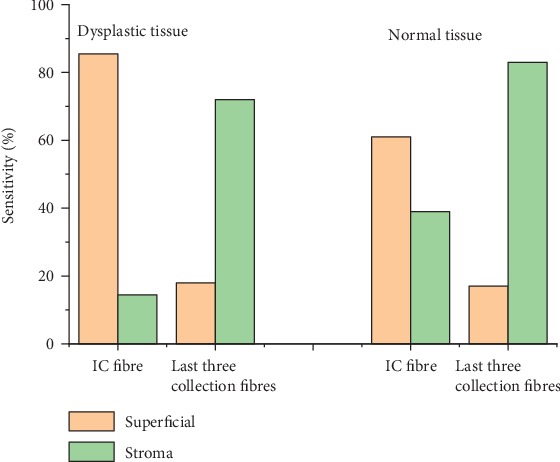
Sensitivity to superficial and stromal layers in MTF_2 resulted from both normal and dysplastic tissue models.

Depending on the results obtained above, the simulation was repeated on both tissue models at a tilting angle of 25° after fixing the illumination fibre to be vertical to the tissue surface and using it as an IC fibre. The fluorescence photons collected from the last three fibres were summed up, and the results of this simulation can be seen in Figure 8.

Results of the dysplastic model showed that the sensitivity of the superficial layers obtained from the IC fibre was about 85.5%, while the sensitivity to the stromal layers obtained by the last three tilted collection fibres was about 72%.

The results of the normal model showed 61% sensitivity to the superficial layers obtained by the IC fibre and 82% sensitivity to the stromal layers obtained by the last three collection fibres.

A comparison between the results of a normal and a dysplastic tissue revealed that the total number of the fluorescence photons collected by the IC fibre in case of a dysplastic tissue was 1.5 times higher than the number collected from normal tissue. This is due to the high sensitivity this fibre has to the superficial layers that exhibit increased fluorescence intensity during cancerous alternations. On the contrary, the number of fluorescence photons collected by the three last fibres in case of normal tissue was 1.42 times higher than the numbers in case of dysplastic ones. This can be explained by the high sensitivity these fibres have to the stromal layers that exhibit decreased fluorescence intensity during cancerous alternations. Overall, the amount of photons collected by the last three collection fibres is small for both types.

### 3.4. MDF Probe

**Figure 9:**
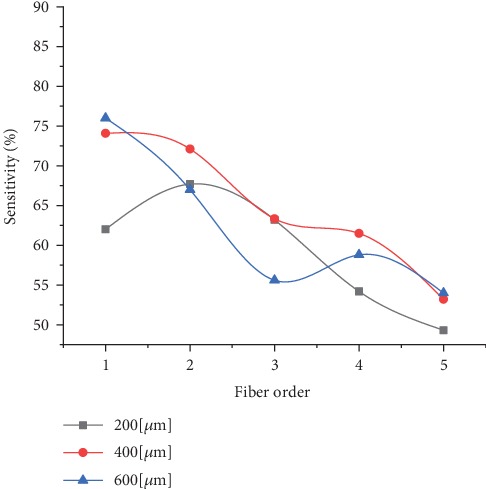
Sensitivity to stromal layers using (MDF).

For the MDF probe, the results indicated that the sensitivity to the stromal layer increased as the collection fibre diameter increased and when the distance between the collection and illumination fibres decreased. As can be seen from Figure 9, the best stromal sensitivity value was approximately about 75% when using the first collection fibre that was set to 600 or 400 *μ*m.

**Figure 10:**
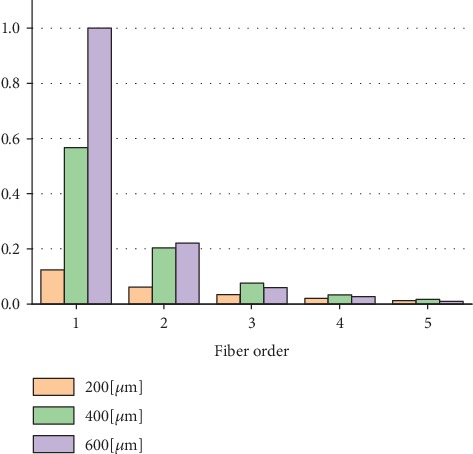
Normalized number of fluorescence photons detected at each collection fibre using (MDF).

However, the amount of fluorescence photons detected increased rapidly with increasing the collection fibre diameter and with decreasing the separation distance between the collection and illumination fibre as presented in Figure 10.

Thus, fibres with 600 *μ*m diameters that are located near the illumination fibres represent the best ones in achieving the highest sensitivity to the stroma layers and the highest amount of fluorescence photons detected.

On the other hand, none of the fibres provided good sensitivity to the superficial layers.

The same explanation of the results provided in the SF paragraph can apply here as well.

### 3.5. The Suggested Geometry

In order to provide a simple probe that does not apply tilting angles and bevelled fibres, this geometry was suggested based on the results obtained from the SF and MDF geometries.

A geometry that is a combination of these two configurations was believed to take advantage of each of them, that is, the high sensitivity to superficial layers by SF and the high sensitivity to the stromal layers provided by MDF.

**Figure 11:**
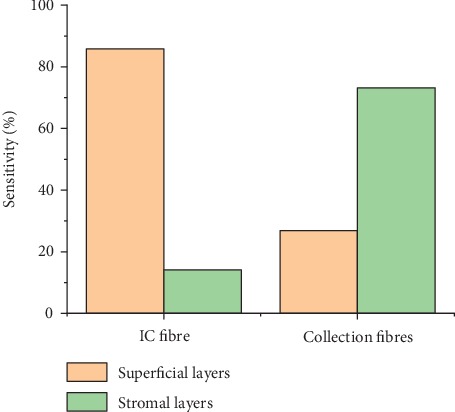
Sensitivity to the superficial and stromal layers using the suggested geometry on the dysplastic tissue model.

**Figure 12:**
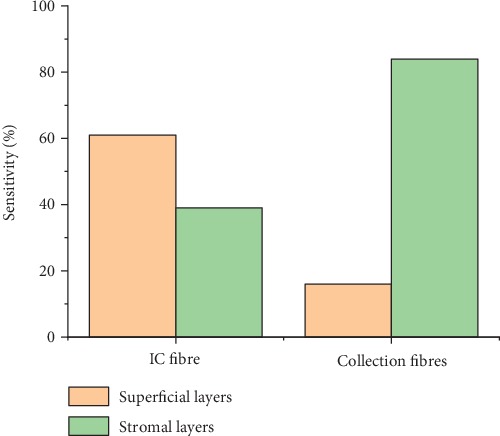
Sensitivity to the superficial and stromal layers using the suggested geometry on the normal tissue model.

Running the Monte Carlo simulation with the suggested geometry on a dysplastic epithelial tongue tissue resulted in a superficial sensitivity of 86% achieved by the IC fibre that was set to 200 *μ*m. It also resulted in stromal sensitivity of 72% achieved by the surrounding fibres that were set to 600 *μ*m each as seen in Figure 11, while the results obtained from the normal tongue indicated stromal sensitivity of 84% achieved by the collection fibre and superficial sensitivity 62% achieved by the IC fibre as seen in Figure 12.

The differences in the results might be due to the fluorescence changes associated with cancer progression mentioned earlier, where the fluorescence intensity increases in superficial layers and decreases in stromal layers during tumour alternations.

Fluorescence intensity collected using this geometry was compared when applying to the normal and dysplastic tissues. The results showed that the fluorescence intensity collected using the IC fibre from the dysplastic tissue is 1.59 times higher than that collected from the normal one, while the fluorescence signal collected by the collection fibres from a normal tissue was 2.13 times higher than that collected from the dysplastic tissue.

Table 2 presents a final comparison between the results obtained by all geometries. A comparison between the suggested geometry and the MTF_2 shows that the results are comparable. However, the total number of detected stromal fluorescence photons in the suggested geometry is higher than the number detected using the MTF_2.

As we have seen, the two most commonly used fibre probe geometries for florescence spectroscopy (SF and MTF) were compared. It can be concluded that both probes exhibit comparable superficial sensitivity. However, MTF probes may provide a higher discriminating feature compared to the SF probe. That is, the MTF has an enhanced ability to distinguish dysplasia and carcinoma in oral epithelium tongue tissue. This finding is of great importance not only for identifying early-stage lesions but also for utilizing the probe as a tool to facilitate targeted guidance of tissue biopsy that would result in shorter surgical procedures. However, it is also important for the probe to be sensitive to both the superficial and stromal layers. For this purpose, we suggested a geometry that achieved 86% sensitivity to fluorescence signal emerging from the superficial layers and 72% sensitivity to that from the stromal layer. These sensitivity values are higher than those in previous studies [19]. It is noteworthy to say that the discrimination between the normal and dysplastic tissue can be achieved using this geometry.

## 4. Conclusions

In summary, this work attempted to suggest a new configuration of optical probe for measuring autofluorescence signals from tongue epithelial tissue taking into consideration the benefits and drawbacks of previous designs.

Practically, further studies to put the suggested geometry in practice could provide more insight into its capability to be used as a noninvasive technique in detecting precancerous lesions in the oral cavity.

Results of this simulation study, particularly the suggested geometry, can be used to design a simple optical fibre probe for enhanced detectability of precancer transformations within the epithelium layer of the tongue. This study could also be extended further to explore the efficiency of these fibre probes for enhancing the depth-limited selectivity of fluorescence measurements emerging from other epithelium layers in the oral cavity.

## Figures and Tables

**Table 1:** Optical properties of tissue layers in normal and dysplastic tongues. FE is the fluorescence efficiency which is the multiplication of the quantum yield and fluorophore absorption coefficient, and fluorescence intensity is the normalized relative fluorescence intensities based on quantitative analysis of the confocal fluorescence images [22].

Layers	Normal tongue			Dysplastic tongue		
	Excitation *λ* = 350 nm	Emission *λ* = 420 nm	Fluorescence intensity	Excitation *λ* = 350 nm	Emission *λ* = 420 nm	Fluorescence intensity
Superficial						
*μ*_*a*_ (cm^−1^)	4.2	3	0.12	4.2	3	0.3
*μ*_*s*_ (cm^−1^)	204	175		204	175	
FE	0.09			0.24	3	
Intermediate						
*μ*_*a*_ (cm^−1^)	4.2	3	0.26	4.2	3	0.4
*μ*_*s*_ (cm^−1^)	66	78		66	78	
FE	0.01			0.144		
Basal						
*μ*_*a*_ (cm^−1^)	4.2	3	0.8	4.2	3	0.8
*μ*_*s*_ (cm^−1^)	320	155		2561	155	0.8
FE	0.38			0.12		
Superficial stroma						
*μ*_*a*_ (cm^−1^)	15	6.22	0.86	15	6.	0.3
*μ*_*s*_ (cm^−1^)	320	270		256	270	
FE	0.38			0.12		
Deep stroma						
*μ*_*a*_ (cm^−1^)	15	21.5	0.86	15	21.5	0.7
*μ*_*s*_ (cm^−1^)	320	270		320	270	
FE	0.38			0.12		

**Table 2:** Comparison between the results obtained by all geometries (the best results obtained by each geometry regarding the superficial and stromal layers).

	SF		MDF		MFT_1		MTF_2		Suggested geometry	
	100 *μ*m diameter	900 *μ*m diameter	The first fibre of 200 *μ*m	The first fibre of 600 *μ*m	The first collection fibre at *B* = 50°	Last three fibres at *B* = 25°	IC fibre	Last three collection fibres	IC fibre	Surrounding fibre
Superficial sensitivity	92%		38%		76%		85.5%		86%	
Stroma sensitivity		55%		76%		83%		72%		72%

## Data Availability

The data used to support the findings of this study are available from the corresponding author upon request.

## References

[1] Bagan J., Sarrion G., Jimenez Y. (2010). Oral cancer: clinical features. *Oral Oncology*.

[2] Schwarz R. A., Richards-Kortum R. R., Gillenwater A. M., Wong B. F., Ilgner J. (2016). Fluorescence and reflectance spectroscopy for detection of oral dysplasia and cancer. *Biomedical Optics in Otorhinolaryngology*.

[3] Patel M., Gomes A., Ruderman S. (2014). Polarization gating spectroscopy of normal-appearing duodenal mucosa to detect pancreatic cancer. *Gastrointestinal Endoscopy*.

[4] Gupta N., Gupta R., Acharya A. K. (2017). Changing trends in oral cancer - a global scenario. *Nepal Journal of Epidemiology*.

[5] Wilder-Smith P., Holtzman J., Epstein J., le A. (2010). optical diagnostics in the oral cavity: an overview. *Oral diseases*.

[6] Bailey M. J., Verma N., Fradkin L. (2017). Detection of precancerous lesions in the oral cavity using oblique polarized reflectance spectroscopy: a clinical feasibility study. *Journal of Biomedical Optics*.

[7] Schwarz R. A., Gao W., Redden Weber C. (2009). Noninvasive evaluation of oral lesions using depth-sensitive optical spectroscopy. *Cancer*.

[8] Nieman L. T., Jakovljevic M., Sokolov K. (2009). Compact beveled fiber optic probe design for enhanced depth discrimination in epithelial tissues. *Optics Express*.

[9] Zhu C., Liu Q., Ramanujam N. (2003). Effect of fiber optic probe geometry on depth-resolved fluorescence measurements from epithelial tissues: a Monte Carlo simulation. *Journal of Biomedical Optics*.

[10] Nieman L. T., Kan C. W., Gillenwater A., Markey M. K., Sokolov K. (2008). Probing local tissue changes in the oral cavity for early detection of cancer using oblique polarized reflectance spectroscopy: a pilot clinical trial. *Journal of Biomedical Optics*.

[11] Drezek R., Sokolov K., Utzinger U. (2001). Understanding the contributions of NADH and collagen to cervical tissue fluorescence spectra: modeling, measurements, and implications. *Journal of Biomedical Optics*.

[12] Pavlova I., Sokolov K., Drezek R., Malpica A., Follen M., Richards-Kortum R. (2003). Microanatomical and biochemical origins of normal and precancerous cervical autofluorescence using laser-scanning fluorescence confocal microscopy. *Photochemistry and Photobiology*.

[13] Lagarto J. L., Credi C., Villa F. (2019). Multispectral depth-resolved fluorescence lifetime spectroscopy using SPAD array detectors and fiber probes. *Sensors*.

[14] Pfefer T. J., Agrawal A., Drezek R. A. (2005). Oblique-incidence illumination and collection for depth-selective fluorescence spectroscopy. *Journal of Biomedical Optics*.

[15] Arifler D., Schwarz R. A., Chang S. K., Richards-Kortum R. (2005). Reflectance spectroscopy for diagnosis of epithelial precancer: model-based analysis of fiber-optic probe designs to resolve spectral information from epithelium and stroma. *Applied Optics*.

[16] Gamm U. A., Hoy C. L., van Leeuwen - van Zaane F. (2014). Extraction of intrinsic fluorescence from single fiber fluorescence measurements on a turbid medium: experimental validation. *Biomedical Optics Express*.

[17] Gillenwater A., Jacob R., Richards-Kortum R. (1998). Fluorescence spectroscopy: a technique with potential to improve the early detection of aerodigestive tract neoplasia. *Head & Neck*.

[18] Inaguma M., Hashimoto K. (1999). Porphyrin-like fluorescence in oral cancer. *Cancer*.

[19] Pavlova I., Weber C. R., Schwarz R. A. (2008). Monte Carlo model to describe depth selective fluorescence spectra of epithelial tissue: applications for diagnosis of oral precancer. *Journal of Biomedical Optics*.

[20] Welch A., van Gemert M. (2011). *Optical-Thermal Response of Laser-Irradiated Tissue*.

[21] Liu Q., Zhu C., Ramanujam N. (2003). Experimental validation of Monte Carlo modeling of fluorescence in tissues in the UV-visible spectrum. *Journal of Biomedical Optics*.

[22] Pavlova I., Williams M., el-Naggar A., Richards-Kortum R., Gillenwater A. (2008). Understanding the biological basis of autofluorescence imaging for oral cancer detection: high-resolution fluorescence microscopy in viable tissue. *Clinical Cancer Research*.

[23] Prahl S. A Monte Carlo Model of Light Propagation in Tissue.

